# Vibratory Ball Milling of Solid‐State Flame‐Retardant Polyelectrolyte Complex for Use in Polymer Emulsions

**DOI:** 10.1002/marc.202500442

**Published:** 2025-09-16

**Authors:** Dallin L. Smith, Kathleen Floyd, Margaret J. Karim, James Batteas, Jaime C. Grunlan

**Affiliations:** ^1^ Department of Chemistry Texas A&M University College Station Texas USA; ^2^ Department of Mechanical Engineering Texas A&M University College Station Texas USA; ^3^ Department of Materials Science and Engineering Texas A&M University College Station Texas USA

**Keywords:** latex, milling, poly (sodium phosphate), polyallylamine, vertical flame testing

## Abstract

Vibratory ball milling reduces the particle size of a polyallylamine, poly(sodium phosphate) containing polyelectrolyte complex (PEC) by an order of magnitude for use as a flame‐retardant additive in a polymer emulsion to achieve self‐extinguishing behavior and a V‐0 rating with UL 94 flame testing. This demonstrates a new approach for using solid PECs as flame‐retardant additives.

## Graphical abstract

Vibratory ball milling reduces the particle size of a solid polyelectrolyte complex (PEC) by an order of magnitude, enabling its use as a flame‐retardant additive in a polymer emulsion to achieve self‐extinguishing behavior. This work demonstrates a new approach for using solid PECs as flame‐retardant additives.

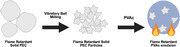

## 1 Introduction

Polymer latexes are suspensions of polymer particles (typically < 1 µm in diameter) stabilized with surfactant [1]. Above the minimum film‐forming temperature (MFFT), the solvent (e.g., water) evaporates, and particles coalesce into a coherent film. While the mechanism of film formation has been one of debate [1, 2, 3], these films are ubiquitous in residential and commercial contexts as decorative and protective coatings that prolong the lifetime of their substrate [4]. Emulsion polymer binders are commonly homopolymers or copolymers of vinyl acetate, methacrylic and acrylic esters, or styrene [5]. For example, Elmer's Glue‐All is a well‐known aqueous adhesive that contains primarily poly(vinyl acetate) (PVAc) and trace amounts of additives (e.g., polyvinyl alcohol and surfactant). Industrially, styrene‐butadiene copolymers are frequently used in carpet backing [6, 7]. In many contexts where these polymers are used (e.g., transportation, construction, and electronics), flammability is a concern, and flame‐retardant materials are used or added [8]. This is especially true for carpet and other flammable textiles used in home furnishings [9].

Polyelectrolyte complexes (PECs) have emerged as environmentally‐benign flame retardant (FR) treatments or additives for a wide range of flammable polymers [10]. They have been applied for fire protection on wood [11], carbon fiber‐reinforced polymers [12], and polyurethane foam [13], using layer‐by‐layer assembly, or on paper [14], fabrics [15], and polystyrene [16] in a single step. In addition to aqueous solutions, solid PECs have been processed (extruded) like thermoplastics, but this requires plasticization [17, 18] (with water or salt) or modification [19, 20]. Due to high levels of crosslinking when dry, PECs are generally brittle and infusible, so they are not amenable to melt processing on their own [19, 21]. Even so, PECs have been successfully melt‐blended as an FR additive with both polylactic acid (PLA) [22] and ethylene‐vinyl acetate (EVA) [23] and, more recently, additively manufactured via vat photopolymerization [24]. Processing PECs in the solid state typically involves suppressing complexation through pH control, lowering the solvent dielectric constant, or using salts for ionic shielding [21]. While these approaches are valuable, additional avenues should be explored to unlock the potential of PECs in non‐plasticizing environments.

Vibratory ball milling is regarded as a low‐cost, environmentally‐benign, and scalable technique used to grind powders into fine particles or perform mechanochemical reactions [25]. In vibratory ball mills, the jar containing milling medium (e.g., stainless steel spheres) and the sample is shaken at a high frequency, and the forces (shear, impact, attrition, and compression) transferred from the medium pulverize the sample particles [25, 26]. However, milling is more often used for the synthesis [27] or degradation [28, 29] of polymers (e.g., polystyrene or poly(ethylene terephthalate) rather than mechanical grinding. While there are reports of vibratory ball milling PEC solutions [26, 30], there are none of a solid complex. By reducing particle size, PECs are more likely to be a feasible additive in an emulsion.

## 2 Results and Discussion

In this study, a PEC composed of poly(allylamine hydrochloride) and poly(sodium phosphate) is added to an aqueous emulsion of PVAc to impart flame retardancy. To reduce particle size, the PEC is first ground on a vibratory ball mill, which is believed to be the first instance of this being done. To mitigate settling while the PVAc dries, a small amount of xanthan gum is added as a rheology modifier. Xanthan gum (XG) is a common food additive that acts as a thickener and stabilizer [31], but has no effect on the flammability of dried PVAc, which burns aggressively. The PEC, on the other hand, intumesces and significantly reduces the fire hazard of PVAc by preventing dripping and attenuating burn times. This strategy demonstrates a new way to use PECs as a flame retardant. While this example serves as an early prototype, the approach could be optimized for many industrial applications on a variety of substrates, such as back coatings on textiles.

**FIGURE 1:**
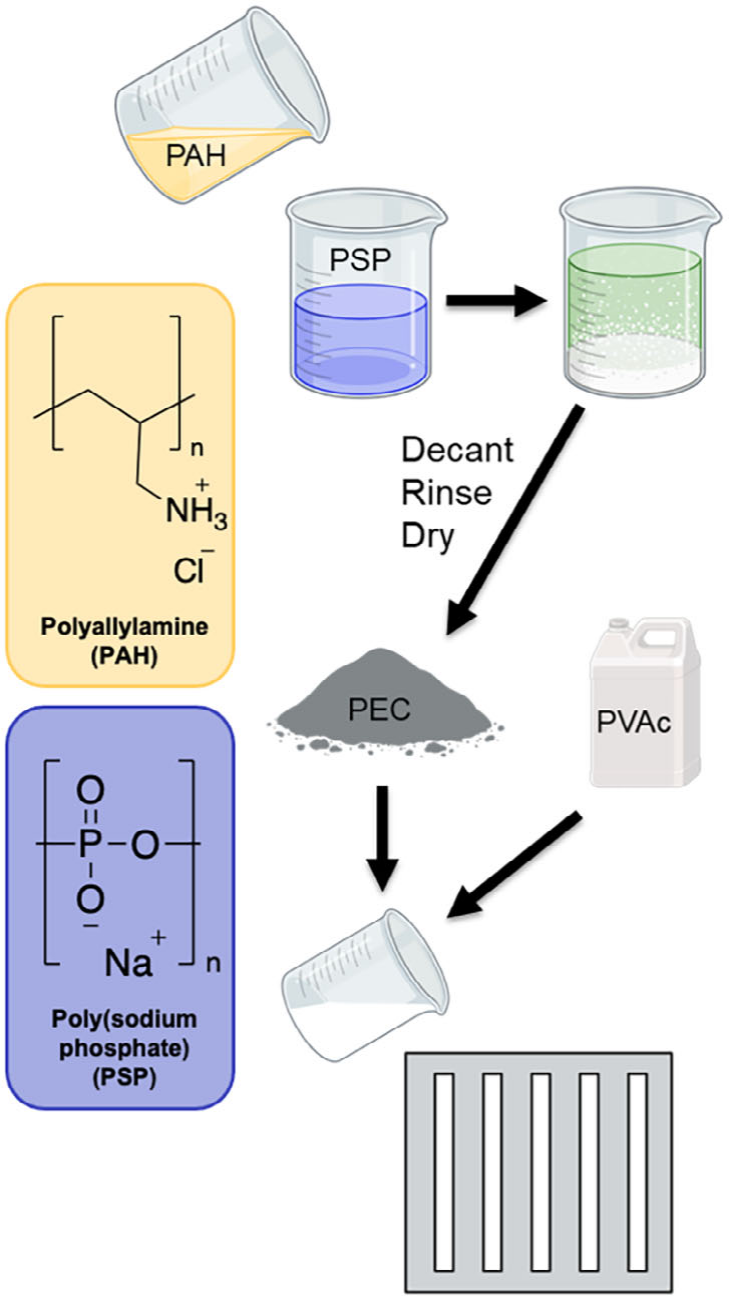
PEC formation, purification, and combination with PVAc before being poured into a Teflon mold. Not pictured: PEC being dried, ground, and vibratory ball milled.

The PEC is prepared by combining equimolar (based on repeat unit molar mass) solutions of aqueous PAH and PSP at their natural pHs (∼3.5 and ∼6.5, respectively). The advantage of this procedure is that both polyelectrolytes are charged when combined, since PSP is a strong polyelectrolyte and PAH has a pK_a_ of ∼9. Because of this, no additional ions are added from the addition of acid or base to adjust pH. Counter ions (Cl^−^ from PAH and Na^+^ from PSP) expelled via complexation are extracted by DI water, which is frequently exchanged. The obtained precipitate is dried on Teflon heat transfer paper at 70°C, ground, and vibratory ball milled with a stainless steel ball to reduce particle size. The PEC is then blended into the PVAc emulsion and poured into a Teflon mold, which is placed in an oven at 30°C for 24 h, then transferred to a sealed box with desiccant for an additional 24 h to obtain rectangular samples. A summary of this process is shown in Figure 1, and a more detailed description can be found in the Supporting Information.

**FIGURE 2:**
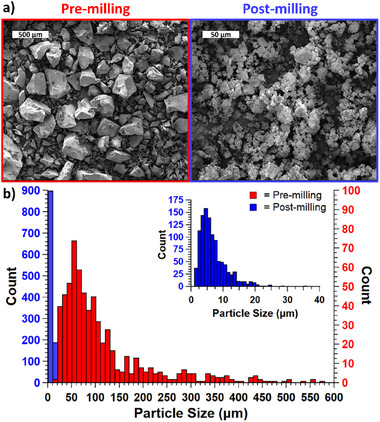
(a) SEM images of PEC particles before and after 10 min of milling. Note the scale bars differ by an order of magnitude. (b) Histogram of particle sizes before and after 10 min of vibratory ball milling.

Prior to milling, PEC particle diameters observed via SEM vary from 10 to 658 µm, with an average of 118 ± 103 µm. The blocky and faceted shape of the particles is fairly consistent. After 10 min of milling, a few large particles remain, but the average diameter is reduced by more than an order of magnitude, down to 7 ± 5 µm (Figure 2b). The largest observed diameter is 35 µm. Longer milling times (30 min, 60 min) were initially explored but showed little additional benefit (Figure S1). Generally, it is understood that particle size and distribution stabilize as a dynamic equilibrium between aggregation and fracture is attained [32]. In this case, their shape also becomes more spherical, and these smaller PEC particles tend to aggregate into clusters with a popcorn‐like appearance (Figure 2a). It is likely that stable particle shape is influenced by the surface area to volume ratio. Vibratory ball milling could destabilize the PEC by creating new surfaces via fracture and disrupting ionic interactions, leading to unbalanced surface charges and charge pairing [33]. Accumulation into clusters could be caused by the instability of small particles, but literature in this space (non‐reactive grinding of solid organics) is sparce. Further work to provide a satisfactory explanation of this observation is beyond the scope of this work, but additional studies focused on understanding the relevant mechanisms are needed and would be beneficial to the PEC community.

**FIGURE 3:**
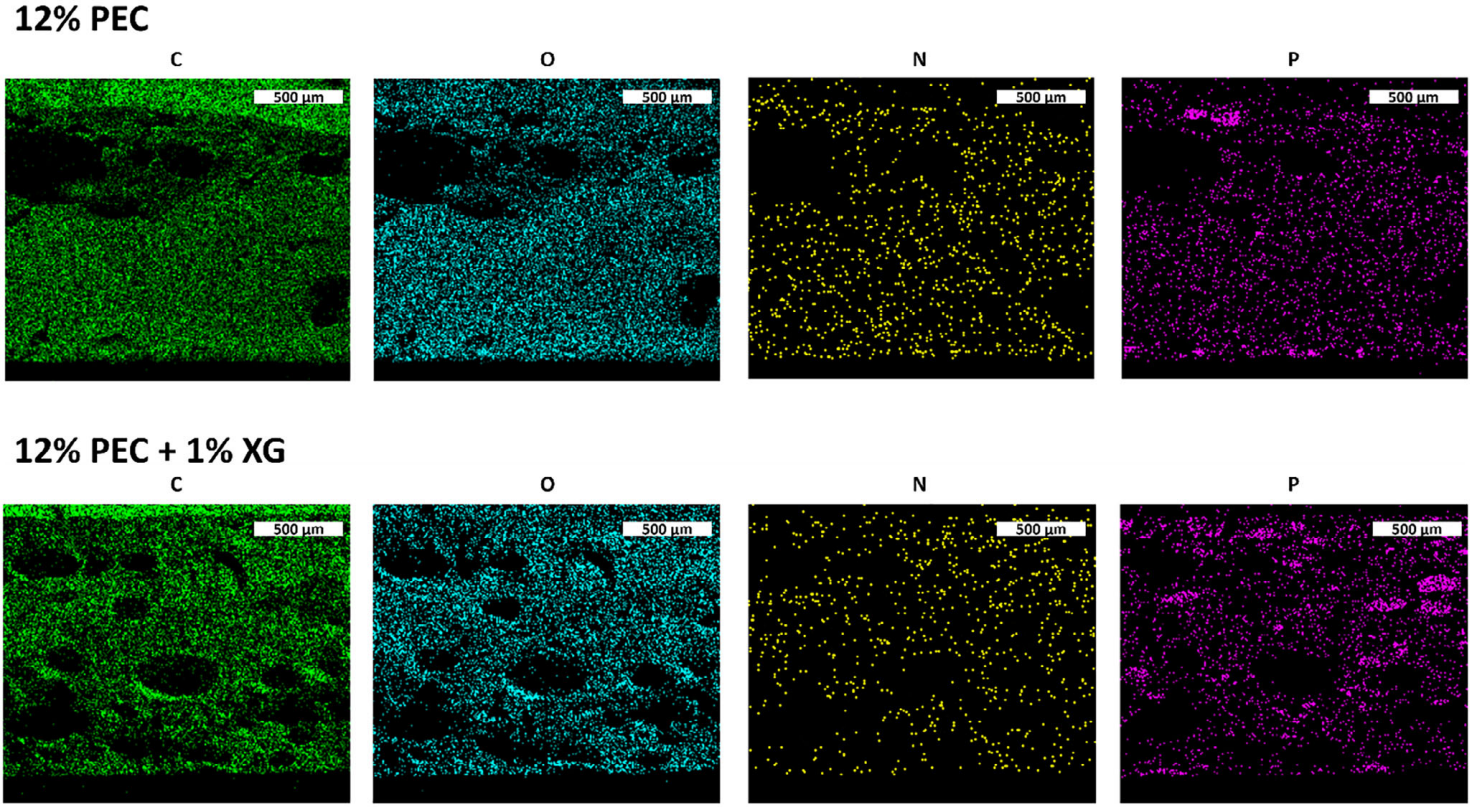
SEM‐EDS maps of PVAc cross‐sections with PEC and XG, showing distribution of carbon, oxygen, nitrogen, and phosphorus. Black domains are the result of air voids in the cross‐section.

Energy dispersive X‐ray spectrometry (EDS) mapping elucidates the distribution (or spatial accumulation) of certain elements in a sample. As the PEC powder is comprised entirely of carbon, oxygen, phosphorus, and nitrogen, there is a homogenous distribution of each element before and after milling (Figure S2). In other words, there are virtually no pockets of individual polyelectrolyte (PAH or PSP) ― where there is one element, there are the others. Nitrogen and phosphorus from the PEC also appear evenly distributed throughout the dried PVAc cross‐section, with or without the addition of 1 wt.% XG (Figure 3). Based on visual observations that PEC particles were settling to the bottom of the PVAc film during drying, it was anticipated that in the absence of XG a higher concentration of P and N would be found at the bottom of the cross‐section, but that does not seem to be the case. In reality, XG may benefit N and P distribution, but EDS may not be sensitive enough to quantify this across samples. Nonetheless, the fire performance of PVAc with 12 wt.% PEC is improved with the addition of 1 wt.% XG.

The UL 94 test is a widely used standard evaluation of polymer flammability [34, 35] in which a sample is exposed twice to a methane flame, and the burn (including dripping) is observed. Ratings ranging from V‐0 (best) to V‐2 (worst) prohibit complete sample consumption. V‐0 and V‐1 ratings are differentiated by total burn time, but flaming drips (indicated by ignition of underlying cotton) are not allowed for either rating. PVAc samples with or without 1 wt.% XG are completely consumed with a long (>50 s) after flame time, and they drip flaming polymer onto the cotton below, resulting in no rating (Table 1). The addition of 12 wt.% PEC eliminates the initial burn time (t_1_) altogether and reduces the second (t_2_), but more importantly, no melt dripping occurs because of charring. This set of 5 samples is rated V‐1 due to one sample having t_2_ = 17 s, but otherwise meets the criteria for V‐0. The combination of 12 wt.% PEC and 1 wt.% XG further reduces t_2_ and achieves a V‐0 rating, probably due to improved dispersion of the PEC particles. Despite EDS element maps, the appearance of samples after UL 94 testing (Figure S3) and higher viscosity, as observed in rheological studies (Figure S4), support this assertion. Without XG, greater conformational change occurs from burning due to a larger gradient of PEC throughout the sample thickness.

**TABLE 1:** UL 94 results for *n* = 5 of each sample type.

Sample	Total t_1_ (s)	Total t_2_ (s)	Flaming drops? (Yes or No)	Rating
PVAc	263	―	YYYYY	Not Rated
1% XG	263	―	YNYYY	Not Rated
12% PEC	0	23	NNNNN	V‐1^a^
12% PEC + 1% XG	0	7	NNNNN	V‐0

^a^ V‐1 rating is caused by a single sample with t_2_> 10 s. Total after flame time (t_1_+t_2_) is still well under the limit (50 s) for V‐0.

**FIGURE 4:**
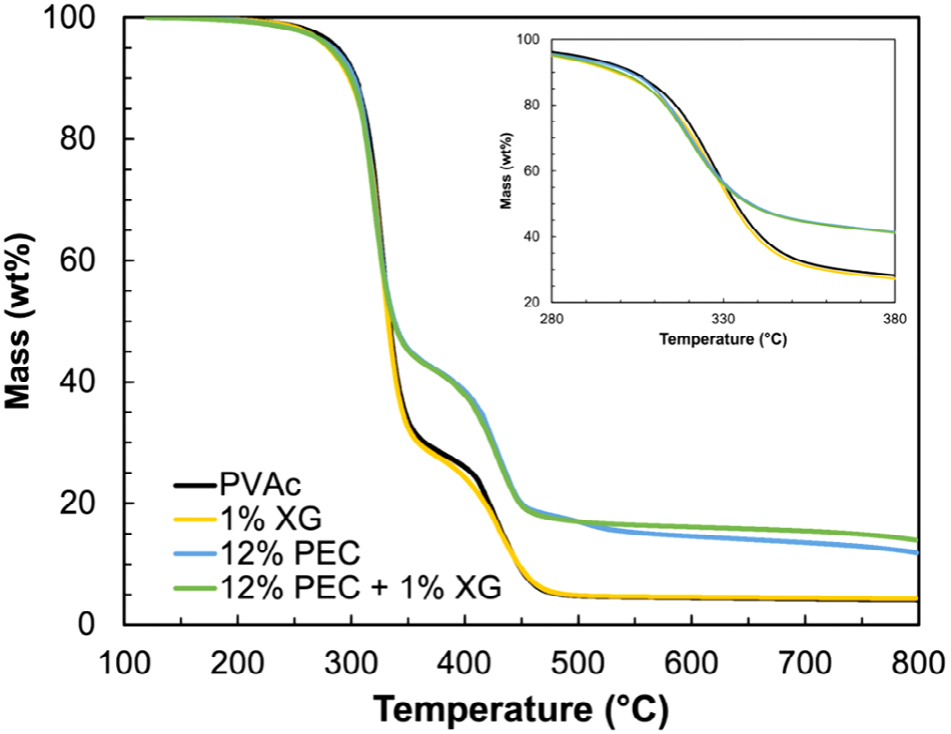
Thermal degradation curves for PVAc samples, with and without additives from 100°C to 800°C. The primary degradation event is shown in the inset (280°C–380°C).

Thermal stability of PVAc with and without additives was investigated with thermogravimetric analysis (TGA). In an inert atmosphere, PVAc mass loss begins around 300°C and peaks at 329°C (Figure 4), which is almost entirely due to gaseous acetic acid generation [36]. A second event occurs around 430°C, possibly from chain scission [37, 38]. Unsurprisingly, the addition of 1 wt.% XG has no effect on the degradation of PVAc. When 12 wt.% PEC is added, a similar curve is obtained, except thermal degradation peaks slightly earlier (∼10°), which is typical for intumescent flame retardants [15]. Furthermore, residue mass at 800°C increases by 200% (from 4% to 12%). This is a result of improved charring facilitated by the phosphorus in the PEC [39]. The PEC itself only yields 22% residue mass (Figure S5), so the improved residue mass of PVAc with 12 wt.% PEC is not merely a weighted average of the two components. PAH/PSP systems have previously been shown to cause intumescence even in non‐carbon‐rich systems, as PSP will promote char formation [15, 40]. The inclusion of 1 wt.% XG with the PEC offers marginal additional improvement to the residue mass (from 11% to 13%) and shows no thermal benefit for neat PVAc.

## 3 Conclusions

The particle size of a polyelectrolyte complex was reduced from ∼100 to ∼10 µm with just 10 min of vibratory ball milling for use as a flame retardant additive in an aqueous poly(vinyl acetate) emulsion. Milling of solid PEC powders is underexplored in the literature, but this work demonstrates its applicability to organic polymeric materials. PVAc burns aggressively, but 12 wt.% PEC additive largely eliminates fire risk and, in combination with 1 wt.% xanthan gum, a V‐0 rating is achieved in UL 94 testing. Similarly, TGA reveals a 200% increase in residual mass at 800°C. This specific system serves as a model for the promising use of PECs as a solid flame retardant additive in a liquid matrix, such as a latex. Modifications can be made to the milling process (e.g., ceramic media and higher frequency) to achieve even smaller particle sizes. In combination with other rheology modifiers, even better distribution (and performance) could be achieved.

## Author Contributions

**Dallin L. Smith**: conceptualization, investigation, methodology, writing – original draft, writing – review & editing, visualization. **Kathleen Floyd**: investigation, methodology, visualization, writing – original draft. **Margaret J. Karim**: investigation, writing – review & editing. **James Batteas**: resources, writing – review & editing. **Jaime C. Grunlan**: conceptualization, resources, supervision, writing – review & editing.

## Conflicts of Interest

The authors declare no conflicts of interest.

## Supporting information




**Supporting File**: marc70063‐sup‐0001‐SuppMat.docx.

## Data Availability

The data that support the findings of this study are available in the supplementary material of this article.
