# Supplementary material for: Vibratory Ball Milling of Solid‐State Flame‐Retardant Polyelectrolyte Complex for Use in Polymer Emulsions
Source: Macromol Rapid Commun. 2025 Sep 16;46(22):e00442. doi: 10.1002/marc.202500442 (PMC12631506; doi:10.1002/marc.202500442)
Supplement: Supplementary file 1 — Supporting File: marc70063‐sup‐0001‐SuppMat.docx. [file MARC-46-e00442-s001.docx]

**Supplementary Information**

**Vibratory Ball Milling of Solid State Flame Retardant Polyelectrolyte Complex for Use in Polymer Emulsions**

*Dallin L. Smith^a^, Kathleen Floyd^a^, Margaret J. Karim^a^, James Batteas^a^, and Jaime C. Grunlan^abc*^*

^a^ Department of Chemistry, Texas A&M University, College Station, TX 77843

^b^ Department of Mechanical Engineering, Texas A&M University, College Station, TX 77843

^d^ Department of Materials Science and Engineering, Texas A&M University, College Station, TX 77843

^*^ Corresponding author

**Table of Contents**

| **Content** | **Page** |
| --- | --- |
| Materials and Methods | S3-4 |
| SEM images of PEC powder with various milling times | S4 |
| SEM-EDS maps of PEC powder showing distribution of carbon, oxygen, nitrogen, and phosphorus | S5 |
| Post-UL-94 images of PVAc samples with PEC. Additional sample contortion is present without 1% XG (left), as well as a larger difference between sides of the sample, because of PEC settling during sample preparation. Top and bottom refer to the sides of the sample in the mold during preparation. | S5 |
| Rheology data of PVAc with and without additives | S6 |
| TGA curves for PEC and its components | S6 |

**Materials and Methods**

*Materials*

Elmer’s Glue-All ® was used as the poly(vinyl acetate) (PVAc) emulsion and acquired from Office Depot (Boca Raton, FL, USA). Poly(allylamine hydrochloride) (PAH) (Mw = 15,000 g·mol^-1^) was purchased from Beckmann-Kenko (Bassum, Germany) and used as received. Xanthan gum from *Xanthomonas campestris* and poly(sodium phosphate) (PSP, crystalline, 96%) were purchased from MilliporeSigma (Burlington, MA) and used as received. Solutions were made with 18 MΩ deionized (DI) water.

*Polyelectrolyte Complex Preparation*

Two equal volume solutions of 0.25M (based on repeat unit; PAH-94 g·mol^-1^, PSP-102 g·mol^-1^) PAH and PSP were separately prepared in DI water and combined. Precipitate immediately formed, and the mixture was stirred on mechanical rollers to allow total complexation to occur. After 30 minutes, the mixture was allowed to settle, and the supernatant was discarded and replaced with fresh DI water. This process was repeated several times until the conductivity of the supernatant read <10 µS/cm with a handheld conductivity meter (Traceable®, VWR, Radnor, PA). The PEC pieces were then spread onto nonstick Teflon heat transfer paper (Amazon, Seattle, WA, USA) and dried in a 70 °C convection oven overnight. The dried PEC was then ground into a fine powder using a coffee grinder (Hamilton Beach, Glen Allen, VA, USA) and dried overnight once more.

*Milling*

Stainless steel jars (25 mL, Retsch) were cleaned with distilled water, ethanol, and dried using N_2_ gas. Jars were then loaded with 2 g of PEC powder and a single stainless steel ball (15 mm diameter, ~13.4 g, Retsch). Next, PEC powder was milled in a Retsch MM 400 operating at 30 Hz for 10 min. Powder was removed and an additional 2 g of powder was added to each jar and milled for 10 min. This process was repeated until all powder had been milled.

*Polymer Composite Preparation*

Additives (xanthan gum and/or milled PEC) were stirred into the emulsion in a plastic beaker and then the mixture was poured into a machined Teflon mold measuring 125 mm x 13 mm x 3 mm. To achieve consistent sample thickness, a razor blade was scraped across the mold to remove excess mixture. The mold was placed in an oven at 30 °C for 24 hours and then transferred into a sealed box with desiccant for another 24 hours. At this point, the individual samples were removed from the mold but returned to the desiccant box for 72 hours to finish drying prior to any testing.

*Characterization*

Vertical burn testing was performed in a UL 94 chamber (Fire Testing Technology, UK) according to ASTM D3801. Thermogravimetric analysis was performed on a TGA 55 from TA Instruments (New Castle, DE, USA) under a 60 mL·min^-1^ sample purge flow and 40 mL·min^-1^ balance purge flow of nitrogen gas. Following a 20 minute isothermal hold at 120 °C to remove adsorbed water, ~20 mg samples were heated at 10 °C·min^-1^ to 800 °C. PEC powders were prepared for imaging by depositing a 10- or 15-nm thick Au layer by sputter-coating (Cressington Scientific Instruments, UK). Preliminary SEM images (Figure S1) were acquired with a beam voltage of 30 kV on a Vega instrument (Tescan Orsay Holding, Czech Republic) and the remainder of SEM images were acquired with a beam voltage of 2, 5, or 10 kV on a FEI Quanta 600 instrument. An Oxford EDS detector was used for EDS mapping with a 10kV beam. Subsequent particle size analysis was performed with ImageJ software. The viscosity and rheological properties of the samples were measured following a flow sweep procedure using 40 mm parallel plates on a Discovery Hybrid Rheometer (HR-2, TA Instruments, New Castle, DE).


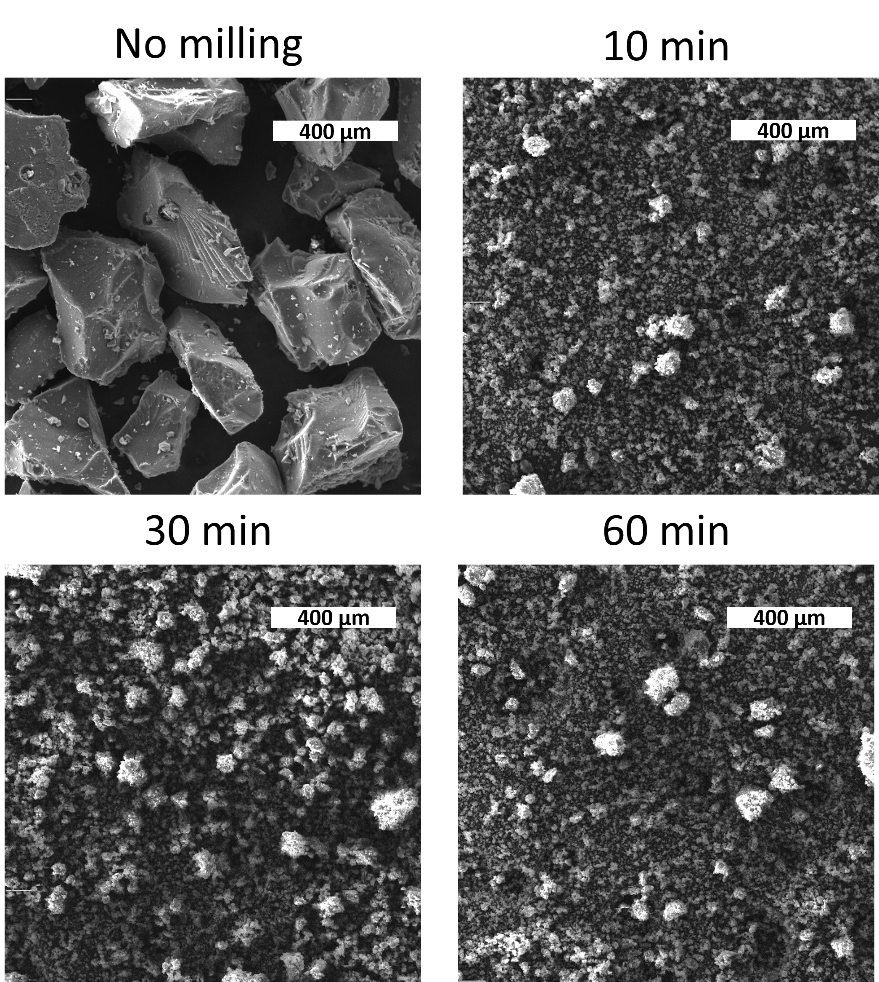


**Figure S1.** SEM images of PEC powder with various milling times.


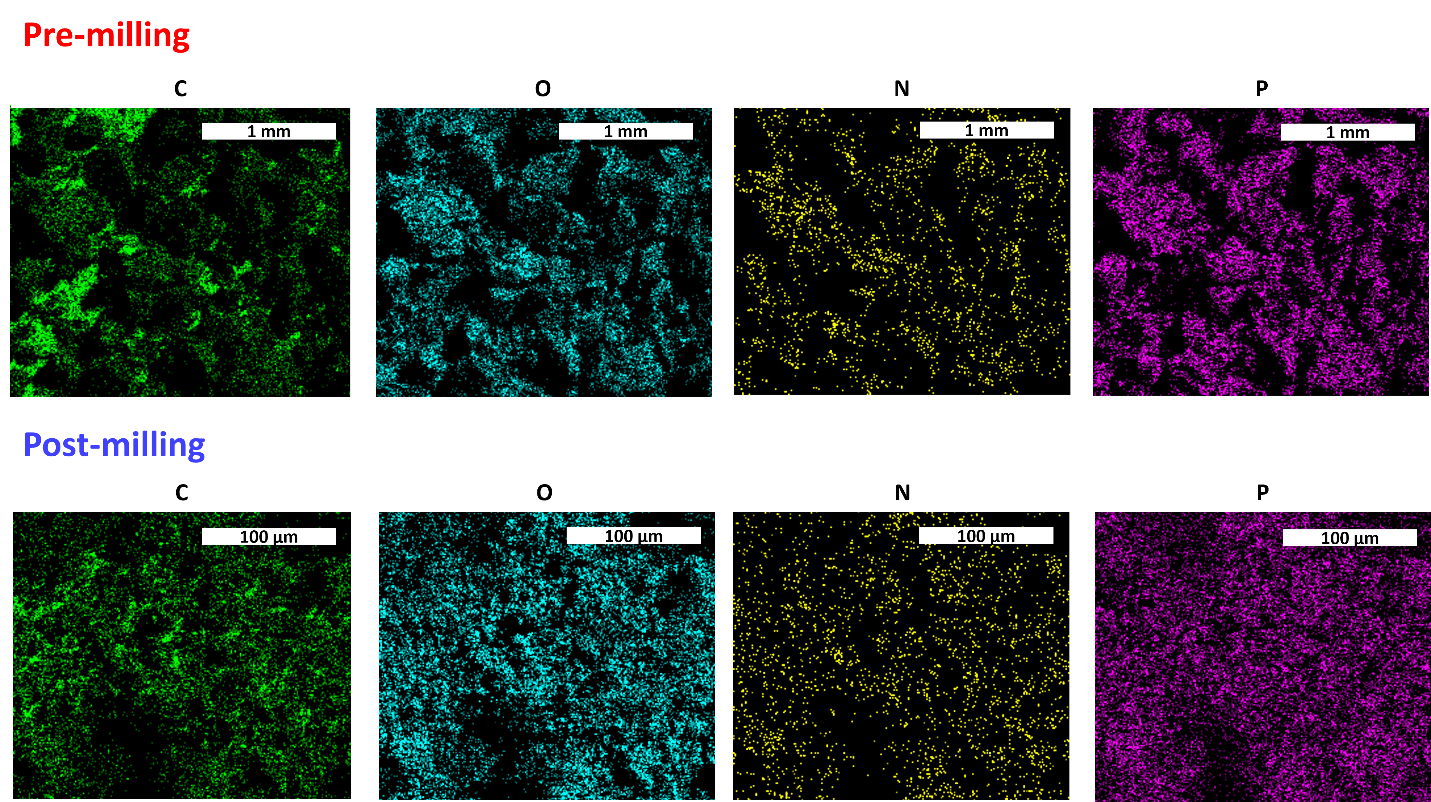


**Figure S2.** SEM-EDS maps of PEC powder showing distribution of carbon, oxygen, nitrogen, and phosphorus.


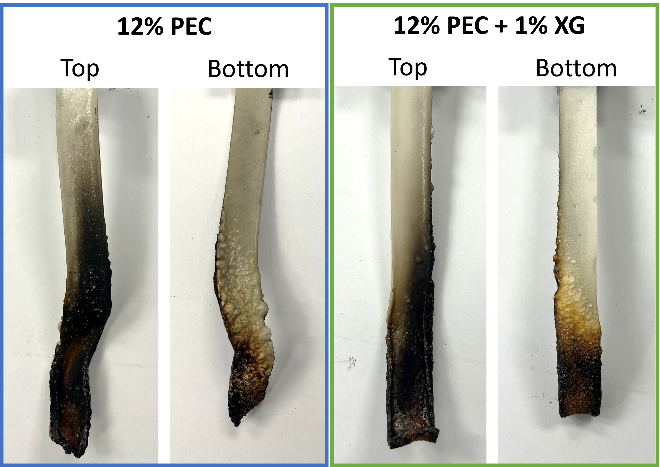


**Figure S3.** Post-UL 94 images of PVAc samples loaded with PEC. Additional sample contortion is present without 1% XG (left), as well as a larger difference between sides of the sample. This is due to modest PEC settling during sample preparation. Top and bottom refer to the sides of the sample in the mold during preparation.


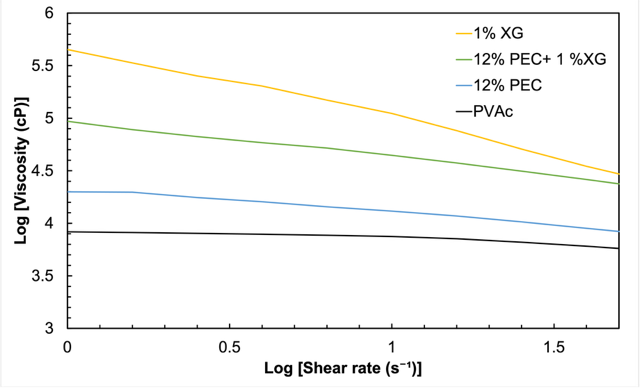


**Figure S4.** Logarithmic plot of viscosity as a function of shear rate for PVAc with and without additives.


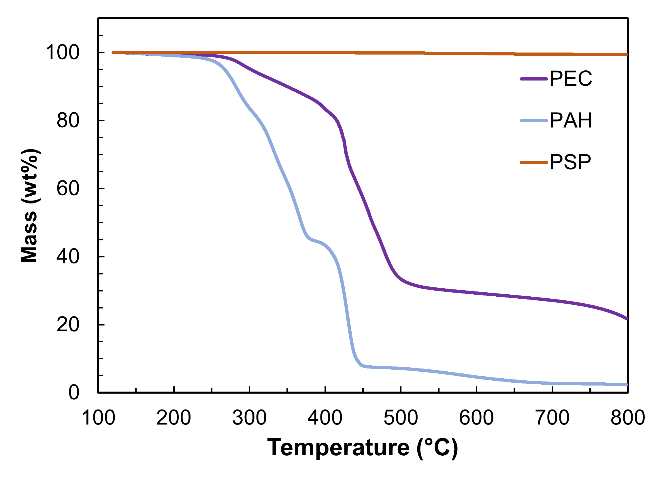


**Figure S5.** TGA curves for PEC and its individual components.
